# The evolution of HIV self-testing and the introduction of digital interventions to improve HIV self-testing

**DOI:** 10.3389/frph.2023.1121478

**Published:** 2023-02-14

**Authors:** Alex Emilio Fischer, Musaed Abrahams, Luke Shankland, Samanta Tresha Lalla-Edward, Vinodh A. Edward, John De Wit

**Affiliations:** ^1^ Aviro Health, Cape Town, South Africa; ^2^ Department of Interdisciplinary Social Science, Public Health, Utrecht University, Utrecht, Netherlands; ^3^ Ezintsha, Faculty of Health Sciences, University of the Witswatersrand, Johannesburg, South Africa; ^4^ The Aurum Institute, Johannesburg, South Africa; ^5^ School of Health Sciences, College of Health Sciences, University of KwaZulu-Natal, Durban, South Africa

**Keywords:** HIV self-screening, digital health, mobile health (mHealth), HIV self-testing (HIVST), HIV, digital intervention

## Abstract

HIV self-testing (HIVST) complements traditional HIV testing programmes by removing barriers and increasing access to testing for key populations, and digital interventions have been developed for HIVST to improve the testing and linkage to care experience for users. The first HIVST kit was proposed in 1986, but it took 10 years for the home sample collection (HSC) HIVST to become available and another 16 years for rapid diagnostic test HIVST to be approved by the Federal Drug Administration. Since then, studies have shown high usability and performance of HIVST, which led the World Health Organization formally recommending HIVST in 2016, and currently almost 100 countries have incorporated HIVST into their national testing strategy. Despite the popularity, HIVST present challenges around pre-and post-test counselling, as well as the ability to report results and link users to care, and digital interventions for HIVST have been introduced to address these challenges. The first digital intervention for HIVST was introduced in 2014 and showed that digital interventions could be used to distribute HIVST kits, report results and link users to care. Since then, dozens of studies have been conducted, which have validated and expanded on these early findings, but many were pilot studies with small sample sizes and lacked the standardization of indicators required to aggregate data across platforms to prove impact at scale. For digital interventions for HIVST to be championed for scale-up, they must continue to show measurable impact at larger scales, while still maintaining and standardizing data security and integrity.

## Introduction

Individuals can quickly learn their HIV status, independent of a healthcare facility, by collecting and testing their own specimen (blood drop from finger prick or an oral swab) with a variety of different HIV self-testing (HIVST) kits (1). HIVST can improve traditional HIV counselling and testing programs by removing barriers associated with stigma and time to access traditional testing, while also promoting frequent testing which may lead to earlier diagnosis and treatment of HIV (2). Although these benefits may be well known now (3), it took decades of research and policy shaping for HIVST to reach this state. In 1986, Elliott Millenson first proposed the idea of using home-based HIVST (4) and now, three and a half decades later, more than 10 million HIVST kits are being distributed each year (5).

Progress over the first 25 years was slow due to a lack of knowledge and policies for HIVST (4, 6), however once the first rapid diagnostic test (RDT) HIVST kit became commercially available in 2012 (7), this allowed for research to be conducted into the usability and performance of HIVST, creating a strong body of evidence. This body of evidence now consists of 32 randomly controlled trials (RCTs) and over 150 values and preference studies, which shows high usability, acceptability and feasibility, in a variety of demographics and regions, while maintaining linkage to care rates, especially in key populations. These outcomes have led to the WHO formally recommending HIVST, and nearly 100 countries adopting them into their national HIV strategies (1, 3).

HIVST has become an effective way to complement existing HIV testing strategies, especially for key populations, however they still present a few challenges for users and healthcare systems in general (1). One main challenge is the lack of appropriate pre-test and post-test counselling (8), while another is that the usage of each self-test cannot be verified or tracked, so not all positive cases are appropriately linked to care (9, 10). To address these challenges, digital interventions for HIVST have been introduced in a variety of ways, including apps, websites and messaging platforms (11–13), and there is now a growing body of evidence that supports digital interventions for HIVST (14). This descriptive perspective will present the evolution of HIVST, including the current challenges, then explore how digital interventions for HIVST are beginning to address these challenges.

## The evolution of HIVST

Laboratory based HIV testing was first made available in 1985, and in many regions testing was introduced with legislation to protect people that tested positive from accidental disclosure and discrimination (15, 16). This legislation also introduced requirements for pre- and post- test counselling, consent to test and how HIV status could be reported, dictating how an HIV status was documented on medical records (4). While these policies were developed to protect the tester and people living with HIV, requirements like reporting positives by name to confidential registries and the need for face-to-face counselling inadvertently hampered innovative testing approaches like HIVST, as it could not comply with these obligations (15).

At that stage, there was inadequate information surrounding HIVST available to advise policy building, so understandably policies were shaped by the concerns of policy makers, surrounding the legal, ethical and social issues that could have potentially occurred from self-testing (17). Legal concerns for HIVST included the inability for lay-people to correctly conduct the test, leading to false positives or false negatives that could spur litigation, while ethical and social concerns included psychological distress, and downstream effects, that may accompany a positive diagnosis outside a health facility. For example, in 1985, before life-saving ARV treatment, a man committed suicide in San Francisco after learning of his HIV positive status. During the first public hearing on HIVSTs, activists distributed copies of this person's obituary as a cautionary tale (4).

**Figure 1:**
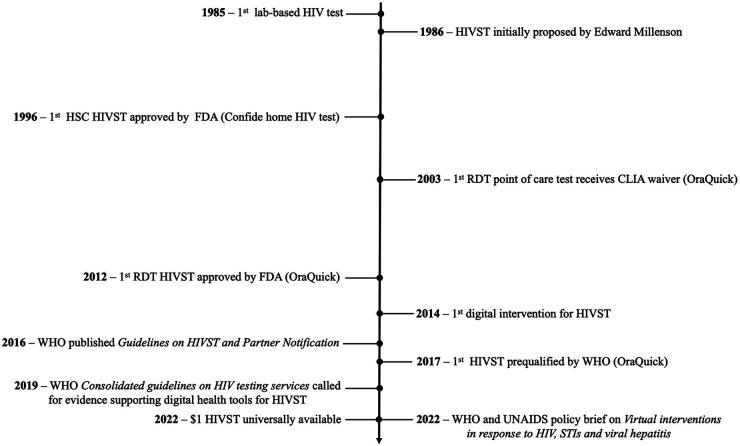
Timeline of HIVST and digital interventions for HIVST. FDA, United States Food and Drug Administration; HIV, human immunodeficiency virus; HIVST, HIV self-testing; HSC, home sample collection; STI, sexually transmitted infection; UNAIDS, The Joint United Nations Programme on HIV/AIDS; WHO, World Health Organisation.

In 1996, with increasing availability of HIV treatment, the United States Food and Drug Administration (FDA) approved the first take-home HIVST kit, the Confide home HIV test by Direct Access Diagnostics (see Figure 1 for a complete timeline of HIVST evolution). Confide home HIV test was a home sample collection (HSC) test, which required a user to collect their own blood sample, mail it to a laboratory for analysis, then call a toll-free number a week or two later for their results and the appropriate post-test counselling (16). The HSC tests were marketed directly to end-users and during the first year of availability, almost 175,000 HSC tests were conducted, with no reports of suicide associated with the testing (16). Other studies have mentioned the possibility of social harm, but none have presented any evidence of suicide or harm related to self-testing (6, 17, 18).

Although HSC tests broke the home testing barrier, users still had to wait weeks before learning their result, and the HSC tests required blood samples from a finger prick, which proved difficult for some users (16). To address these challenges, a new type of HIV test, the RDT, was developed, which could be conducted with an oral fluid specimen or blood, and the results were revealed in minutes, not weeks (19). In 2003, OraQuick became the first RDT to be waived by the Clinical Laboratory Improvements Amendments Law, which was the first step towards becoming approved for HIVST. The OraQuick waiver did not allow for in-home testing like HSCs, but it did allow for point of care testing at doctors' offices, instead of just traditional laboratory settings (20).

RDTs proved to be easy to use and the short wait times meant that 30%–40% of testers in public facilities were no longer being lost to follow-up before learning their results (19). There were, however, concerns with accuracy, as the Centers for Disease Control and Prevention reported in a Morbidity and Mortality Weekly Report that one testing programme in New York City experienced clusters of false positives, which totaled over 400 from 2005 to 2008, exceeding the specificity confidence interval of the manufacturer (21). The cause of these false positive clusters was not discovered, but it seemed to be an isolated incident, as no large-scale studies reported sensitivity (ability to detect true positives) or specificity (ability to filter out true negatives) values that fell outside the manufacturer's specifications.

The threat of inaccurate results may have delayed the HIVST approval process, but in 2012, the FDA approved OraQuick as the first ever over the counter RDT, joining HSCs as a faster option for HIV home testing (7). Two years after OraQuick's FDA approval, a review of over 300 articles was conducted on self-testing, including 49 on HIVST, and the authors concluded that there was very little evidence of any harm (phycological, social or medical) because of HIVST. This review went on to recommend that HIVST programs should be expanded, and not restricted based on the potential fears of harm that self-testing may cause (17).

After the initial FDA approval, the development of HIVST programmes was slow, and as of July 2015, only two countries were implementing HIVST supported by national policies, but after the WHO released their Guidelines on HIV Self-Testing and Partner Notification in 2016 (1), there was a shift. Programmes like HIV Self-Test AfRica (STAR) were launched to study the use of HIVST in low- and middle-income countries (LMICs), and their findings, as well as findings from other studies, began to show the true potential of HIVST (22). A usability assessment with seven different HIVST products (Biosure, Atomo 1, Atomo 2, Calypte, OraQuick, Insti and Chembio), and almost 1,500 untrained users was conducted in South Africa. The assessment reported that 96% of the participants thought the tests were easy to use and felt confident using them unassisted (23). These findings were verified a year later by a study of four HIVST products (Biosure, OraQuick, Insti and Chembio), where 97% of the 3,600 users, gave them high usability scores. Furthermore, this study revealed that the sensitivity and specificity of the tests was 98.2% and 99.8%, respectively, surpassing the performance measures attained during FDA approval (24). This study was also conducted with a minimum sample size of 900 users per HIVST product, allowing the results to be used for WHO prequalification (25).

WHO prequalification is a programme that started in the 1980s as a way for UNICEF to determine whether the vaccines they purchased met appropriate quality standards (25, 26). Since then, the programme has expanded to include the prequalification of pharmaceuticals, including antiretrovirals (ARVs) and *in vitro* devices, including HIVST kits (27). For HIVST kits, the prequalification process includes a review of packaging and instructions for use, evidence from studies on usability and clinical performance by untrained users, and a site visit of the manufacturing facilities (25). In 2017, the WHO recognised OraQuick as the first prequalified HIVST kit, and now there are six HIVST kits that have WHO prequalification, including one that is available for only US$1 (5, 28).

Despite the strong body of evidence leading to a formal recommendation by WHO, affordable WHO prequalified products, and supportive national policies in almost 100 countries, challenges with HIVST still remain (3). HIVST can shift testing away from healthcare facilities, but this shift also removes the traditional pre-test and post-test counselling provided by trained healthcare workers or counsellors (8). Furthermore, the shift away from facilities also creates a challenge around the ability to show that HIVST can create a measurable health impact, which is difficult because each individual self-test cannot be appropriately tracked and not all positive cases are linked to care (9, 10). Digital interventions for HIVST have been proposed to improve HIVST programmes by addressing these challenges and in 2019, the *Consolidated guidelines on HIV testing service*s was published by the WHO, which called for evidence supporting the potential for digital health tools to optimise HIVST. Specifically, the guidelines highlighted demand generation, video-based counselling and facilitating linkage to care as areas where evidence supporting digital interventions for HIVST is needed (29).

## Digital interventions for HIVST

Digital interventions for HIVST are a type of digital health that incorporates digital technology, in the form of telehealth, apps, social media, messaging platforms or the internet, to complement HIVST by addressing the challenges of traditional HIVST programmes (30). These digital interventions have been used to promote and distribute HIVST kits, deliver video counselling, provide instructions for use, and link self-testers to appropriate care, including preventative services, like preexposure prophylaxis, for negative self-testers and ARVs for positive self-testers (14). The very first HSC programs for self-testing in the 1990s required users to call a toll-free number for their HIVST results, where they could also access pre-recorded information about their results, a textbook example of telehealth (31). If that same telehealth programme was released today, it would be considered a digital intervention for HIVST, however this terminology did not exist in the 1990s, and the telephones and recordings may very well have still been analog, not digital.

To the authors' knowledge, the first digital intervention for HIVST that was academically evaluated, with findings published in a peer-reviewed journal was in 2014, within 2 years of the FDA approval of OraQuick. The intervention used a social networking app on smartphones, called Grindr, to increase HIVST by promoting a website that distributed free HIVST kits to men who have sex with men (MSM) in Los Angeles. In two months, nearly 12,000 people accessed the website, which led to 334 requests for HIVST kits, two of which tested positive and were linked to care (32). This study showed potential for digital interventions to monitor public health impact by tracking positives and linkage to care. Since then, dozens of studies have been conducted to validate and expand on these early findings (11, 13, 14, 33, 34).

South Africa is one of the leading implementers of HIVST and building off the findings from the usability and performance assessments of HSTAR in sub-Saharan Africa (22–24), there was a series of compounding studies in the same region that illustrated the development of digital interventions for HIVST (11, 13, 33, 34). The first study focused on the usability, acceptability and feasibility of digital interventions for HIVST, and findings confirmed that users found these digital tools highly usable and acceptable (11). The study observed 300 South African users with no prior HIVST experience, while they conducted OraQuick self-tests, assisted by the Aspect smartphone app, a digital intervention designed to improve the testing and reporting experience for HIVST users. The *Aspect* app walked the user through the instructions for use, the collection and testing of their oral fluid specimen, then the reporting of results to a central database by uploading a picture of their self-test result. Of the 300 users, 296 (98.7%) found it easy to use, with 267 (89.0%) users correctly completing all steps and all but one (299/300; 99.7%) stating they would be willing to use the app again (11).

While the *Aspect* study was conducted under the supervision of a healthcare worker in a facility (11), another smartphone app, Ithaka was introduced, which let a similar sample of users from Johannesburg, South Africa self-test and report results at home, independent of a healthcare facility (13). *Ithaka* was a progressive web app for OraQuick, which expanded from just instructions for use, by adding pre- and post- test counselling, before the user self-reported their results. The pilot included 751 users, which led to 295 (39.3%) receiving counselling and 168 (22.4%) self-reported results, including 14 (8.3%) that reported as HIV positive (13). The *Ithaka* app was also adapted from the original home-based configuration, to complement facility-based HIVST as well. In the facility, visitors could self-test in a booth, guided by the *Ithaka* app, which provided digital instructions, followed by audio visual pre- and post- test counselling, as well as the ability to self-report results. The addition of digitally assisted HIVST with *Ithaka* led to a 25% increase in total testing numbers, without compromising the positivity yield (33).

Another South Africa study that paired OraQuick with a digital intervention for HIVST showed that digital interventions for HIVST could also successfully be used to link self-testers to care. Over 3,000 participants from Cape Town were invited to do traditional HIV testing, supervised digital HIVST at the facility, or unsupervised digital HIVST off-site (34). The digital intervention was *HIVSmart!*, an app that guided users through the instructions for use then linked patients to counselling and care; ARVs for positive self-testers and prevention pathways for negative self-testers. The conventional HIV testing (control) arm linked 98.5% of patients to care. The supervised digital HIVST arm was slightly lower with a 95.7% linkage to care rate and the unsupervised digital HIVST arm was slightly higher than the control with a linkage rate of 99.3% (34).

The above studies (11, 13, 33, 34) were presented for their similar methodologies and progressing outcomes, but these findings have also been validated by independent studies in different regions and populations (14). A recent systematic review of digital interventions for HIVST confirmed that digital interventions could be used to link users to care, by aggregating findings from 12 studies, including studies from Asia, America and Europe. Five of the studies used social media or apps to link patients to care at a rate of 80%–100%, which was more effective than the seven web-based platforms, where only 53%–100% of users were linked to care (14).

This review also revealed one of the main challenges with digital interventions for HIVST, which is the lack of standardisation and cohesion across platforms. For example, linkage to care was not standard across all studies, and varied to include a clinic referral, post-test counselling, confirmatory testing, or ART initiation, depending on the study. This led the authors to suggest the need for a digital health framework focused on the diagnostic outcomes of HIV (14). In 2022, at the International AIDS conference in Montreal, the WHO and UNAIDS released a policy brief on *Virtual interventions in response to HIV, sexually transmitted infections and viral hepatitis*, which provides guidance for incorporating digital interventions into traditional programmes, including HIVST. This document champions the use of digital interventions for HIVST, while also attempting to standardise their implementation and indicators (35).

## Conclusion

Despite challenges around counselling, reporting self-test results and linkage to care, HIVST has grown in use, especially over the past decade, with over 10 million HIVST kits currently distributed each year. Digital interventions for HIVST have been introduced in a variety of ways, and the research examining HIVST interventions chiefly consists of pilot studies that lacked ability to show impact at scale. This shotgun approach has also led to incompatible datasets across different interventions and regions, impeding data harmonisation and intervention scale-up. For digital interventions to realise universal acceptance, they must begin to show measurable, comparable impact at scale, while also maintaining data security and integrity. Future research needs to focus on large-scale implementation, and explore the need for regulatory approval or prequalification of digital interventions for HIVST, as a way to standardise these interventions beyond generic data privacy policies (36).

## Data Availability

The original contributions presented in the study are included in the article/Supplementary Material, further inquiries can be directed to the corresponding author/s.
