# Corrigendum: The evolution of HIV self-testing and the introduction of digital interventions to improve HIV self-testing

**DOI:** 10.3389/frph.2023.1241434

**Published:** 2023-08-01

**Authors:** Alex Emilio Fischer, Musaed Abrahams, Luke Shankland, Samanta Tresha Lalla-Edward, Vinodh A. Edward, John De Wit

**Affiliations:** ^1^ Aviro Health, Cape Town, South Africa; ^2^ Department of Interdisciplinary Social Science, Public Health, Utrecht University, Utrecht, Netherlands; ^3^ Ezintsha, Faculty of Health Sciences, University of the Witswatersrand, Johannesburg, South Africa; ^4^ The Aurum Institute, Johannesburg, South Africa; ^5^ School of Health Sciences, College of Health Sciences, University of KwaZulu-Natal, Durban, South Africa

**Keywords:** HIV self-screening, digital health, mobile health (mHealth), HIV self-testing (HIVST), HIV, digital intervention

A Corrigendum on The evolution of HIV self-testing and the introduction of digital interventions to improve HIV self-testing By Fischer AE, Abrahams M, Shankland L, Lalla-Edward ST, Edward VA and De Wit J. (2023) Front. Reprod. Health 5:1121478. doi:10.3389/frph.2023.1121478

In the published article, there was an error in Figure 1 as published. The third line displays “**1996**—1st HSC HIVST approved by FDA (Home Access HIV)”. The correct third line is “**1996**—1st HSC HIVST approved by FDA (Confide home HIV test)”. The corrected Figure 1. appears below.

**Figure:**
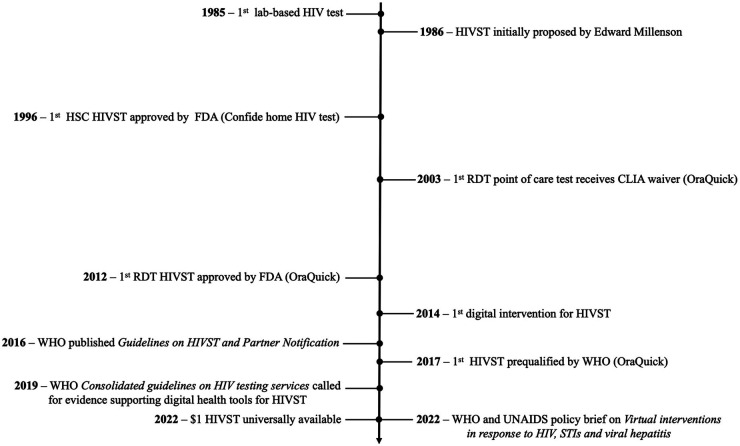


In the published article, there was an error. The first take-home HIVST kit, was “Confide home HIV test by Direct Access Diagnostics”, not the “Home Access HIV test system”, as originally displayed. A correction has been made to **The evolution of HIVST**, paragraph 3. This sentence previously stated:

“In 1996, with increasing availability of HIV treatment, the United States Food and Drug Administration (FDA) approved the first take- home HIVST kit, the Home Access HIV test system (see Figure 1 for a complete timeline of HIVST evolution). Home Access HIV was a home sample collection (HSC) test, which required a user to collect their own blood sample, mail it to a laboratory for analysis, then call a toll-free number a week or two later for their results and the appropriate post-test counselling (16).”

The corrected sentence appears below:

“In 1996, with increasing availability of HIV treatment, the United States Food and Drug Administration (FDA) approved the first take-home HIVST kit, the Confide home HIV test by Direct Access Diagnostics (see Figure 1 for a complete timeline of HIVST evolution). Confide home HIV test was a home sample collection (HSC) test, which required a user to collect their own blood sample, mail it to a laboratory for analysis, then call a toll-free number a week or two later for their results and the appropriate post-test counselling (16).”

The authors apologize for these errors and state that this does not change the scientific conclusions of the article in any way. The original article has been updated.

